# The mating brain: early maturing sneaker males maintain investment into the brain also under fast body growth in Atlantic salmon (*Salmo salar*)

**DOI:** 10.1007/s10682-014-9715-x

**Published:** 2014-06-05

**Authors:** Alexander Kotrschal, Susanne Trombley, Björn Rogell, Ioana Brannström, Eric Foconi, Monika Schmitz, Niclas Kolm

**Affiliations:** 1 Department of Animal Ecology, Evolutionary Biology Centre, Uppsala University, Norbyvägen 18D, 75236 Uppsala, Sweden; 2 Department for Integrative Biology and Evolution (KLIVV), Veterinary University Vienna, Savoyenstrasse 1A, 1160 Vienna, Austria; 3 Department of Organismal Biology, Comparative Physiology, Evolutionary Biology Centre, Uppsala University, Norbyvägen 18A, 75236 Uppsala, Sweden; 4 School of Biological Sciences, Monash University, Clayton, VIC 3800 Australia; 5 Department of Ecology and Genetics, Evolutionary Biology Centre, Uppsala University, Norbyvägen 18D, 75236 Uppsala, Sweden; 6 Department of Zoology/Ethology, Stockholm University, 10691 Stockholm, Sweden

**Keywords:** Brain, Atlantic salmon, Mating strategy, Sneaker, *Salmo salar*, Brain development, Trade-off

## Abstract

It has been suggested that mating behaviours require high levels of cognitive ability. However, since investment into mating and the brain both are costly features, their relationship is likely characterized by energetic trade-offs. Empirical data on the subject remains equivocal. We investigated if early sexual maturation was associated with brain development in Atlantic salmon (*Salmo salar*), in which males can either stay in the river and sexually mature at a small size (sneaker males) or migrate to the sea and delay sexual maturation until they have grown much larger (anadromous males). Specifically, we tested how sexual maturation may induce plastic changes in brain development by rearing juveniles on either natural or ad libitum feeding levels. After their first season we compared brain size and brain region volumes across both types of male mating tactics and females. Body growth increased greatly across both male mating tactics and females during ad libitum feeding as compared to natural feeding levels. However, despite similar relative increases in body size, early maturing sneaker males maintained larger relative brain size during ad libitum feeding levels as compared to anadromous males and females. We also detected several differences in the relative size of separate brain regions across feeding treatments, sexes and mating strategies. For instance, the relative size of the cognitive centre of the brain, the telencephalon, was largest in sneaker males. Our data support that a large relative brain size is maintained in individuals that start reproduction early also during fast body growth. We propose that the cognitive demands during complex mating behaviours maintain a high level of investment into brain development in reproducing individuals.

**Electronic supplementary material:**

The online version of this article (doi:10.1007/s10682-014-9715-x) contains supplementary material, which is available to authorized users.

## Introduction

Brain morphology is highly variable at all taxonomic levels among vertebrates (Jerison 1973; Kotrschal et al. 2013) and explaining this variation continues to be an important question in modern evolutionary biology (Striedter 2005). Theory and empirical data maintain that brain morphology evolves under the balance between positive selection for cognitive ability and the energetic costs of maintaining a larger and more complex brain (Aiello and Wheeler 1995; Darwin 1871; Isler and van Schaik 2006; Jacobs 1996; Jerison 1973; Kotrschal et al. 2013). Comparative analyses and recent experimental data have found support for cognitive benefits of increased relative brain size (Kotrschal et al. 2013), increased neuron density (Haug 1987) and increased size of specific brain regions (Maguire et al. 2006). We adhere to the broad definition of “cognition” as comprising “all mechanisms that invertebrates and vertebrates have for taking in information through their senses, retaining it, and using it to adjust behaviour to local conditions” (Shettleworth 2010). At the same time, given the energetically costly nature of the vertebrate brain, trade-offs between investment into the brain and other costly features of an organism’s biology have long been assumed to be important in generating variation in brain morphology (Aiello and Wheeler 1995; Boogert et al. 2011; Kotrschal et al. 2013; Navarrete et al. 2011; Striedter 2005). Interestingly, despite many decades of interest in the selection pressures for increased brain size and potential trade-offs, experimental data are still scarce regarding the selection pressures that affect brain complexity.

A key-aspect of any organism’s life history is reproduction (Roff 1992). Reproduction is also interesting from the perspective of brain evolution and development since it may assert selection for both increases and decreases in brain size. It has been proposed that cognitive ability is highly important during courtship, mating and parental care (Boogert et al. 2011; Gittleman 1994; Gonzalez-Voyer et al. 2009b; Jacobs 1996; Jacobs et al. 1990). Hence, the requirement of complex behaviours associated with reproduction could select on increased brain size or complexity. At the same time, reproduction is a highly costly feature in any organism (Harshman and Zera 2007; Roff 1992; Williams 1966) and could therefore place important constraints on brain development and evolution (Isler and van Schaik 2009). In support of this, Kotrschal et al. (2013) recently demonstrated a negative association between offspring number and relative brain size in lines of guppies artificially selected for large and small relative brain size.

Sexual maturation is a critical aspect of reproduction and its timing has dramatic effects on most aspects of life-history (Roff et al. 2002). To date, although it is well known that brain morphology and function change dramatically during development, particularly until sexual maturation (Redies and Puelles 2001), almost nothing is known about the relationship between brain development and sexual maturation in an ecological context. Given the cognitive demands of reproduction and the potential trade-off between energetic costs of brain development and reproduction, insights into the relationship between relative brain size and sexual maturation should be informative to reveal the potential selection pressures that act on brain development and evolution.

Here we study the relationship between sexual maturation and plasticity in brain morphology development in Atlantic salmon (*Salmo salar*), a species in which males can adopt either of two mating tactics. Males can stay in the river and sexually mature at a small size (‘early maturing’ males), or migrate directly to the sea and delay sexual maturation until obtaining much larger size (‘anadromous’ males, Jones 1959). The anadromous males are migratory and will defend spawning territories upon return to the river, while the early maturing males adopt a sneaker tactic. As we were interested in the energetic trade-offs in brain development across different mating tactics, we manipulated the potential of energetic acquisition. Hence, we deployed a food ratio treatment by using two different food-regimes: restricted feeding, which closely matches natural feeding levels, and unrestricted (standard hatchery: ad libitum) feeding.

Following this food ratio manipulation, we compared body size, relative brain size and relative brain region volumes across early maturing males, future anadromous males (from now on ‘anadromous’) and females of the two different feeding regimes. To ensure that potential differences are due to the species’ selective past (evolutionary change) and not due to divergent requirements or behaviours of mating tactics during the experiment (phenotypic plasticity), individuals were reared in standard hatchery tanks. These standard hatchery conditions did not provide the early maturing males with sneaking opportunities due to the absence of mature territorial males and spawning females. If sexual maturation and the mating behaviours associated with reproduction have a positive influence on neural development, we predict that early maturing males will have increased relative brain development in relation to other treatments. Alternatively, under a trade-off between sexual maturation and brain size development, we predict that early maturing males will have smaller brain size, particularly under natural feeding levels, than both anadromous males and females. For brain region volumes, it is often difficult to make accurate predictions since the function of the separate brain regions is still only partly understood and because single regions sometimes have multiple functions (Striedter 2005). We therefore avoid making predictions for the brain region volumes and treat this part of the analysis as a prospect to identify the regions of the brain that are most affected by the interaction between growth and sexual maturation.

## Materials and methods

Atlantic salmon parr was provided by the SLU Fishery Research Station in Älvkarleby (60°N, 17°E) hatchery in Sweden. The fish at the hatchery in Älvkarleby originate from the natural salmon population from river Dalälven. Diadromous fish are prevented from following their natural migration route owing to a hydropower dam. Adult salmon migrating upstream are caught with a catching case and transported to a sorting hall, where they are kept and used for artificial breeding. The juveniles are reared at this hatchery and then released as smolts, usually at the age of two years (Petersson et al. 2013). The 1-year old fish for this experiment (n = 3200, mean weight 7.2 g (6.9, 7.7) were randomly distributed into sixteen 1 m^3^ tanks. At this point all fish are immature and the decision to mature or not has not yet taken place. The fish were reared under natural photoperiod, with through flowing water at ambient temperature: The experiment was started in April when water temperatures were about 6 °C. Water temperature steadily increased during spring and summer, reaching temperatures of about 20 °C during June and July. From August onwards, water temperature decreased again and at the end of the experiment in October it was about 11 °C. Two different feeding regimes were started in April where one group was fed salmon feed ad libitum (‘unrestricted feeding’) and one group was fed at a 50 % ration of the control group (restricted feeding; from here on ‘natural feeding level’). Because the here used population is regularly stocked with hatchery-reared animals, hatchery-level (unlimited) food is a part of their evolutionary relevant environment. The difference in food treatment is known to increase the number of early maturing males in the unrestricted compared to the natural feeding treatment due to differences in energy/adiposity levels, which affects maturation propensity (Rowe and Thorpe 1990). The composition of the commercial salmon food (Aller performa MM, Aller Aqua, Denmark) was 54 % protein, 15 % fat and 12 % carbohydrates. Food levels were calculated for optimized hatchery growth in the unrestricted feeding group and calculated growth rates ranged from 1.3 to 3.3 % weight gain per day. In the natural feeding level group growth rates ranged from 0.3 to 1.9 %, which is in the seasonal range observed in wild populations of 1-year old Atlantic salmon parr (Bacon et al. 2005) and also corresponds to the source population of the hatchery fish (Petersson et al. 2013). In the unrestricted fed group mortality was 5.5 % (SE 2.0) and in the natural group 6.6 % (SE 1.56) with no significant differences between treatments. There was no cannibalism observed.

At 19 months of age we euthanized the fish by prolonged exposure to metomidate hydrochloride (Aquacalm, Syndel Co., Vancouver, Canada), quantified their weight and total length, decapitated them and placed their heads in 10 % phosphate buffered formalin (4 % paraformaldehyde). Fish were sexed by visual inspection of the gonads, whereby females and anadromous males show thread-like (non-functional) gonads, but early maturing males show fully developed gonads and running milk. To quantify brain weight and brain region volumes, we removed the brains from the skull and weighed them to the nearest mg. Digital images of the dorsal, ventral, left and right side of the brain were taken through a dissection microscope. For each image the brain was symmetrically positioned such that one hemisphere did not appear larger than the other based on perspective. The widths of six key structures (olfactory bulb, telencephalon, optic tectum, cerebellum, hypothalamus and dorsal medulla) were determined from dorsal and ventral views, whereas brain region lengths and heights were taken from lateral views. Structure volumes were determined following Pollen et al. (2007). For paired structures both sides were measured and the volumes added to give total structure volume. We closely followed Kotrschal et al. (2012c) and obtained brain measures of 297 fish balanced over food treatment, sex and maturation strategy. Three male samples were damaged in the dissection process and therefore excluded (one anadromous male from the natural feeding level, one early maturing male from the natural feeding level, and one anadromous male from the unrestricted feeding treatment).

### Statistical analysis

In order to analyze the effect of sex (three levels: “female”, “early maturing male” and “anadromous male”) and feeding treatment (two levels, “natural level feeding” and “unrestricted feeding”) on the total brain volume and the six brain components we fit individual linear mixed effect models with tank identity and the tank * sex interaction as random effects. Size, sex, treatment and the interaction between sex and treatment were added as fixed effects. We used body length (fork length) as a proxy for body size in the analyses where we controlled for size since this measure is less affected by rapid weight changes associated with sexual maturation. Further, all possible second order interactions between the fixed effects were tested using likelihood ratio tests but were found to be non-significant and hence omitted from the models. Size, brain volume and the volume of the six brain components were also log10 transformed prior to analysis in order to avoid problems associated with allometry. Body size was analyzed using a similar linear model with sex/mating tactic and feeding regime as fixed factors and tank nested under sex as random effect. To correct for multiple testing in the analyses of brain structures we employed a false discovery rate controlling procedure (Benjamini and Hochberg 1995). All models were performed in R Development Core Team (2006).

## Results

**Fig. 1:**
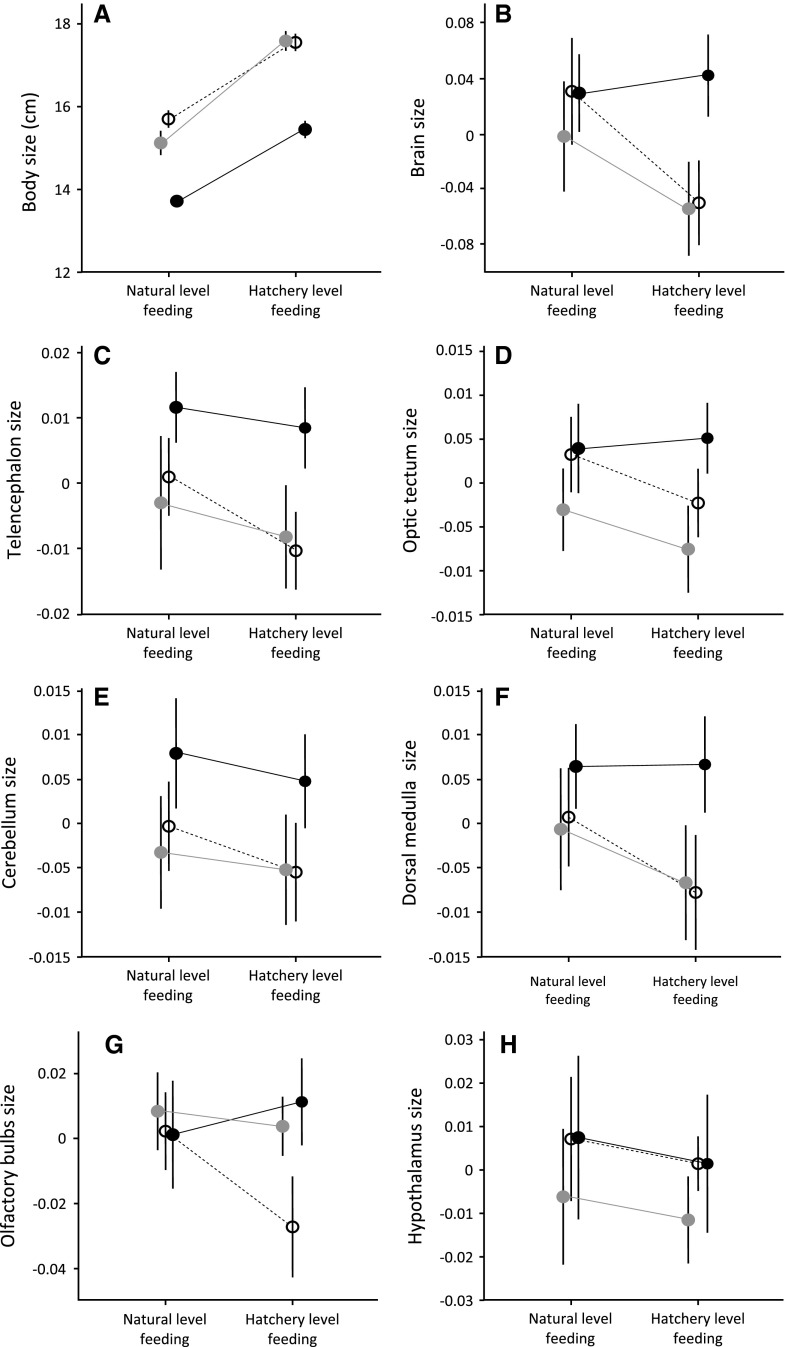
The effects of food availability and growth strategy on somatic and neural growth in male and female Atlantic salmon (*Salmo salar*). **a** Body size [total length (cm) ± SE], **b** relative brain size [weight (g) ± SE], **c**–**h** relative brain structure sizes for fish fed unrestricted (hatchery level) or natural level diets. *Open circles* females, *filled circles* early maturing males, *grey circles* future anadromous males). Shown are the means of linear mixed-effect models controlling for rearing tank

We found a strong effect of feeding level on the proportion of males that matured early (proportion early maturing males in the natural level feeding treatment: 37 %; in the unrestricted feeding treatment: 56 %; *T* test, t_14_ = 7.55, *P* < 0.001). Early maturing males were substantially smaller than anadromous males and females in both feeding treatments (mean body size of early maturing males: natural level feeding: 136.4 mm (95 % CI 133, 141), unrestricted feeding: 154.2 mm (95 % CI 151; 158); body size of anadromous males: natural level feeding: 151.4 mm (95 % CI 146, 156), unrestricted feeding: 175.3 mm (95 % CI 171, 181); mean body size females: natural level feeding: 156.6 mm (95 % CI 152, 161), unrestricted feeding: 176.6 mm (95 % CI 173, 181). The differences in size were significant both across sexes/mating strategies and across feeding treatments (Table 1). Importantly, we did not find any significant interactions between food regime and sexual status, indicating that the increase in body size across food regime was similar across the sexual groupings (Table 1). Relative brain size did not differ between mating tactics and sexes under natural level feeding (Table 1; Fig. 1). However, during the fast body growth in the unrestricted feeding treatment, early maturing males maintained their relative brain size while both anadromous males and females underwent a large reduction in relative brain size, as evidenced by the significant interaction between mating tactic and feeding treatment on relative brain size (sex × treatment interaction: χ^2^ = 8.75, *P* = 0.014, Table 1; Fig. 1b). We detected several differences in the size of separate brain regions across our treatments, sexes, and mating strategies (Table 2; Fig. 1). For example, relative telencephalon, optic tectum, cerebellum and dorsal medulla sizes were generally larger in fish reared on unrestricted feeding (Table 2; Fig. 1). For mating tactics we found that, relative telencephalon, cerebellum and dorsal medulla sizes were larger in maturing males compared to anadromous males and females (Table 2; Fig. 1). Relative optic tectum size was similarly larger in females and early maturing males, but smaller in anadromous males (Table 2; Fig. 1). The olfactory bulbs were not different across groups apart from the much smaller olfactory bulbs in females under unrestricted feeding. Finally, relative hypothalamus size was unaffected by both food treatment and mating tactic (Table 2; Fig. 1).

**Table 1:** Results from linear mixed effect models with body size as dependent variable (left), and for total brain weight as dependant variable and body size as covariate

	Body size			Brain size		
	χ^2^	*df*	*P*	χ^2^	*df*	*P*
Fixed effect						
Food treatment	49.77	1	***	25.62	1	***
Group	146.57	2	***	23.50	2	***
Body size	–	–	–	813.49	1	***
Treatment × group	4.27	2	0.118	8.57	2	0.014
Random effect	Variance			Variance		
Tank	0.14			8.49 × 10^−6^		
Tank × group	0			0		
Residual	1.67			6.23 × 10^−4^		

All variables were log_10_-transformed prior to analysis

*** *P* < 0.001

**Table 2:** Results from linear mixed effect models with separate brain structures as dependent variables

	Telencephalon			Optic tectum			Cerebellum			Dorsal medulla			Olfactory bulbs			Hypothalamus		
	χ^2^	*df*	*P*	χ^2^	*df*	*P*	χ^2^	*df*	*P*	χ^2^	*df*	*P*	χ^2^	*df*	*P*	χ^2^	*df*	*P*
Fixed effect																		
Food treatment	9.98	1	**0.002**	20.24	1	***	15.43	1	***	9.12	1	**0.003**	4.00	1	0.046	1.21	1	0.290
Group	23.44	2	***	6.43	2	**0.040**	14.54	2	***	25.93	2	***	7.17	2	**0.028**	0.82	2	0.664
Body size	117.94	1	***	491.22	1	***	385.59	1	***	352.79	1	***	48.49	1	***	45.91	1	***
Treatment × group	1.37	2	0.504	0.691	2	0.691	0.40	2	0.817	4.21	2	0.122	2.69	2	0.260	0.01	2	0.998
Random effect	Variance			Variance			Variance			Variance			Variance			Variance		
Tank	1.18 × 10^−4^			1.12 × 10^−4^			2.68 × 10^−4^			1.60 × 10^−4^			1.06 × 10^−4^			1.55 × 10^−3^		
Tank × group	1.96 × 10^−15^			1.34 × 10^−19^			1.63 × 10^−4^			9.62 × 10^−5^			1.21 × 10^−19^			1.90 × 10^−3^		
Residual	2.72 × 10^−3^			1.11 × 10^−3^			1.88 × 10^−3^			1.94 × 10^−3^			9.26 × 10^−3^			1.08 × 10^−2^		

All variables were log-transformed prior to analysis

Bold values denote statistically significant effects at α = 0.05 after a controlling for false discovery rate (Benjamini and Hochberg 1995)

*** *P* < 0.001

## Discussion

The two different male mating tactics and the females all increased their body size considerably more during the unrestricted feeding treatment as compared to the natural level feeding. Despite that the increase in body size was close to parallel between early maturing males and anadromous males and females in the unrestricted feeding regime, the early maturing males maintained their relative brain size also under unrestricted feeding while both anadromous males and females decreased their relative brain size as compared to the natural level feeding treatment. We detected several differences in brain region volumes across feeding regimes, mating tactics and sex. Most prominently, irrespective of food availability, the early maturing males showed the relatively largest telencephalon and cerebellum.

Food availability is one of the strongest environmental factors to influence the choice of whether or not to mature early in male salmon. Indeed we found that a higher proportion of males matured early in the unrestricted fed animals. High growth rates (Berglund 1992) and large adiposity stores (Shearer and Swanson 2000) during certain times of the year promote the sneaker strategy choice and therefore a nutrient rich diet fed in abundance increases the number of early maturing male parr. However, there is also a heritable component to this choice, where the actual threshold levels of adiposity and/or growth rates that need to be reached in order for early sexual maturation to commence, are partially genetically determined (for review see Fleming 1996). This means that when food is scarce, less fish will reach these genetically set thresholds levels necessary for investment in sexual maturation and will instead adopt the alternative anadromous phenotype (or mature in freshwater the following year). These threshold levels likely explain much of the individual variation seen, since not all fish of the same size and adiposity will mature.

Our results suggest that sexually mature individuals maintain investment in neural development under fast growth and may provide experimental support for the hypothesis that the behaviours associated with mating require high cognitive ability, that have resulted in a link between maturation and brain size (Boogert et al. 2011; Kotrschal et al. 2012a). Under natural (low) food availability this difference was not apparent, which at first seems to oppose the argument and may suggest that only unnaturally high food levels create the observed effect. However, the population of fish used in this study was for generations regularly stocked with hatchery-reared animals. Hence, unrestricted (hatchery) feeding levels are a part of their evolutionary environment. That only the sexually mature males keep their investment into the brain proportional to somatic growth while the non-mature animals change their investment in relative brain size therefore strongly suggests that a large brain size is vital for sexually mature individuals. The fact that early maturing fish in both feeding treatments showed enhanced telencephalon and cerebellum size, and that the telencephalon, and to some extent also the cerebellum (see below) are the main regions responsible for cognitive abilities in fishes (Broglio et al. 2003), again corroborates the assumption that behaviours associated with mating require high cognitive ability.

But what are the proximate mechanisms behind the observed differences in neural development between early maturing males and anadromous males and females? During this period in nature all juvenile fish are territorial, and thereafter only the early maturing sneaker males continue to defend territories in the river while the anadromous animals form schools during the migration to coastal waters prior to sexual maturation. During mating, the sneaker males approach courting pairs and quickly try to fertilize eggs of egg-laying females. Based on these differences in the behaviour of the two male mating tactics, we identify two, not necessarily mutually exclusive, selection pressures associated with the specific behaviours of sexually mature sneaker males: (1) the cognitive demands of territory defence, and (2) the cognitive demands of the complex mating behaviours of the sneaker males. Since territory defence occurs also in anadromous juvenile Atlantic salmon parr irrespective of sex and mating tactics (Stradmeyer and Thorpe 1987), we propose that it is the latter of these two possibilities: that the complex behaviours associated with mating require the increased neural investment observed in early maturing males. Given the extremely aggressive nature of anadromous males in diadromous salmonids during mating, successful mating by depositing sperm onto the eggs of spawning females by sneaker males require highly precise coordination and timing (e.g. Elliott 1994). We therefore expect strong selection on the precision of sperm release and on avoiding the much larger anadromous males that can otherwise seriously injure a much smaller sneaker male.

Alternatively, the physiological process of sexual maturation may be correlated in timing with brain development. Although we cannot rule out this possibility, empirical evidence from other taxa such as mice and humans suggests that brain development can occur to a surprisingly large degree also after sexual maturation (Casey et al. 2008; Epstein 1979). Furthermore, neurogenesis is known to occur during the entire life span in fish (Zupanc 2001). Because the anadromous males in our study were still immature we can not disentangle whether it is the maturation process per se or the need for coordinating the complex behaviours associated with sneaking tactic that leads to a relatively larger brain in ad lib fed early maturing males and a larger telencephalon in early maturing fish of both feeding groups. However, the fact that a recent study in wild brown trout (*Salmo trutta*) found that precocious (sneaker) males have relatively larger brains than mature anadromous males (Kolm et al. 2009) suggests that sneaking behaviour is generally associated with a relatively larger neural investment. In our experiment the mature males had no opportunity for sneaking behaviour, suggesting that previous selection has generated a developmental link between the brain and the body size that is buffering relative brain size from an environmental factor, food availability.

Selection for fast growth in combination with relaxed selection on brain size in anadromous individuals may additionally have contributed to our results because anadromous fish benefit more from attaining a large body size than sneaker males. First, in females, a large body size allows for large gonads and therefore has a direct impact on fitness by increasing reproductive output (Fleming and Gross 1990). Second, in anadromous males, a large body size confers competitive benefits when establishing a mating territory (Metcalfe et al. 2003). Third, future anadromous fish undergo parr smolt transformation (smoltification), adapting them for a life in the marine environment, and seawater adaptability is highly size-dependent (Jones 1959). Fourth, mortality usually decreases strongly with increasing body size, because the most important aquatic predators are gape-size limited (Sogard 1997). This size-dependant predation pressure is stronger and persists for longer in the marine habitat of the anadromous animals because predators are larger in marine than in freshwater systems (Wootton 1998). Due to those selection pressures, the anadromous individuals may have evolved the capacity to invest surplus energy into growing larger bodies to the expense of their relative brain size. Indeed, previous studies on cichlid fishes (Gonzalez-Voyer et al. 2009a), pinnipeds (Fitzpatrick et al. 2012), bats (Pitnick et al. 2006), carnivores (Gittleman 1994), and primates (Smaers et al. 2012) have demonstrated that the allometric relationship between brain and body can be highly variable at the taxonomic scale of species and above. Building on those studies, we now show that variation in the allometric association between brain and body occurs also at the intraspecific level as a plastic response linked to mating tactic/strategy. This highlights the need to carefully consider evolutionary and plastic changes in both absolute brain size and body size when investigating how relative brain size co-varies with aspects of ecology and life history.

Despite that both neural development and the onset of sexual maturation are both highly energetically costly (Kotrschal et al. 2013; Roff 1992), we did not detect any trade-offs between these processes regardless of feeding level. Instead, as mentioned above, relative investment into brain size and brain structure sizes was greater under natural feeding levels. We note that the lower, natural level feeding treatment fish still received a relatively natural feeding level. Hence, an even harsher low feeding level treatment might have been necessary to yield detectable effects. Moreover, trade-offs can occur at many different levels and our analysis do not provide a complete picture of all potential aspects that could be negatively affected by the maintenance of resources into brain development in the early maturing sneaker males. Hence, it is pre-mature to consider that the relatively large brain size in comparison to body size comes at no cost for sneaker males. Future studies will aim at investigating the full suite of consequences from this shunt of resources into the brain.

Evolutionary variation in separate brain region sizes is high and this variation has been linked to ecology and mating behaviour (e.g. Barton and Harvey 2000; Devoogd et al. 1993; Gonzalez-Voyer and Kolm 2010; Iwaniuk and Nelson 2001; Kotrschal et al. 1998). Moreover, plastic changes in brain regions have been reported before, for instance in relation to social environment (Gonda et al. 2009; Kotrschal et al. 2012b) or physical environment (Kotrschal et al. 2012c). Apart from the relatively larger telencephalon and cerebellum in early maturing males, we detected several more differences in separate brain region volumes among the male tactics, sexes, and feeding treatments. While the hypothalamus remained rather similar in size across all groups, the optic tectum, cerebellum and dorsal medulla were more prominent in early maturing, compared to anadromous males. Moreover, except for the optic tectum, the females’ structures closely followed the anadromous males’ structures. Both optic tectum and cerebellum play important roles in cognition and coordination that can be linked to sneaker male behaviour during mating. That mature males had larger cerebellum contrasts against a previous study on brown trout where anadromous males had larger cerebellum (Kolm et al. 2009). We note that this previous study included anadromous males that had smoltified and returned from the sea while all the fish in the present study were parr. We therefore speculate that the cerebellum size increase in anadromous fish occurs during smoltification, or at least in closer temporal proximity to the sea migration. Because the dorsal medulla is part of the brain stem which controls the autonomous nervous system (Nieuwenhuys et al. 1998) it is feasible that diverse tasks of reproduction, including the innervation of functional gonads, demand a larger dorsal medulla in reproductively active animals such as the early maturing males in this study. Indeed, maturation in fish seems to be associated with an increase in brain stem size (Brandstatter and Kotrschal 1990). Surprisingly, we found that while the olfactory bulbs were relatively similar in size across groups and feeding regimes, they were substantially smaller in hatchery level fed females. Olfactory bulb size is closely linked to olfactory capacity and acuity (Kotrschal et al. 1998). If a decrease in olfactory needs underlies this decrease in olfactory bulb size it may be that a high food environment relaxes the necessity to search for food using this sensory modality. However, why this effect is sex-specific remains enigmatic.

To conclude, we show that early maturing sneaker males maintain their relative brain size also during rapid body growth under unrestricted feeding, while anadromous males and females show a dramatic decrease in relative brain size, despite showing parallel relative increase in body size as compared to the early maturing sneaker males. Maturation therefore concurs with increased investment in neural tissue. We speculate that these results may be generated by the cognitive demands of the complex mating behaviours of sneaker males. Relatively larger brains may give sneaker males an advantage during sneaking and so lead to coevolution of increased investment into neural tissue and maturation. That sneaking is cognitively demanding is further corroborated by relatively larger telencephalon and cerebellum size in sneaker males irrespective of feeding treatment. Our findings are therefore consistent with the hypothesis that maturation, possibly via the cognitive demands of mating behaviour, places high demands on cognitive ability and can be an important engine of brain morphology diversification both at the intra- and interspecific level.

## Electronic supplementary material

Below is the link to the electronic supplementary material.

Supplementary material 1 (PDF 42 kb)
