# Supplementary figures and images for: The mating brain: early maturing sneaker males maintain investment into the brain also under fast body growth in Atlantic salmon (Salmo salar)
Source: Evol Ecol. 2014 Jun 5;28(6):1043–55. doi: 10.1007/s10682-014-9715-x (PMC4459551; doi:10.1007/s10682-014-9715-x)

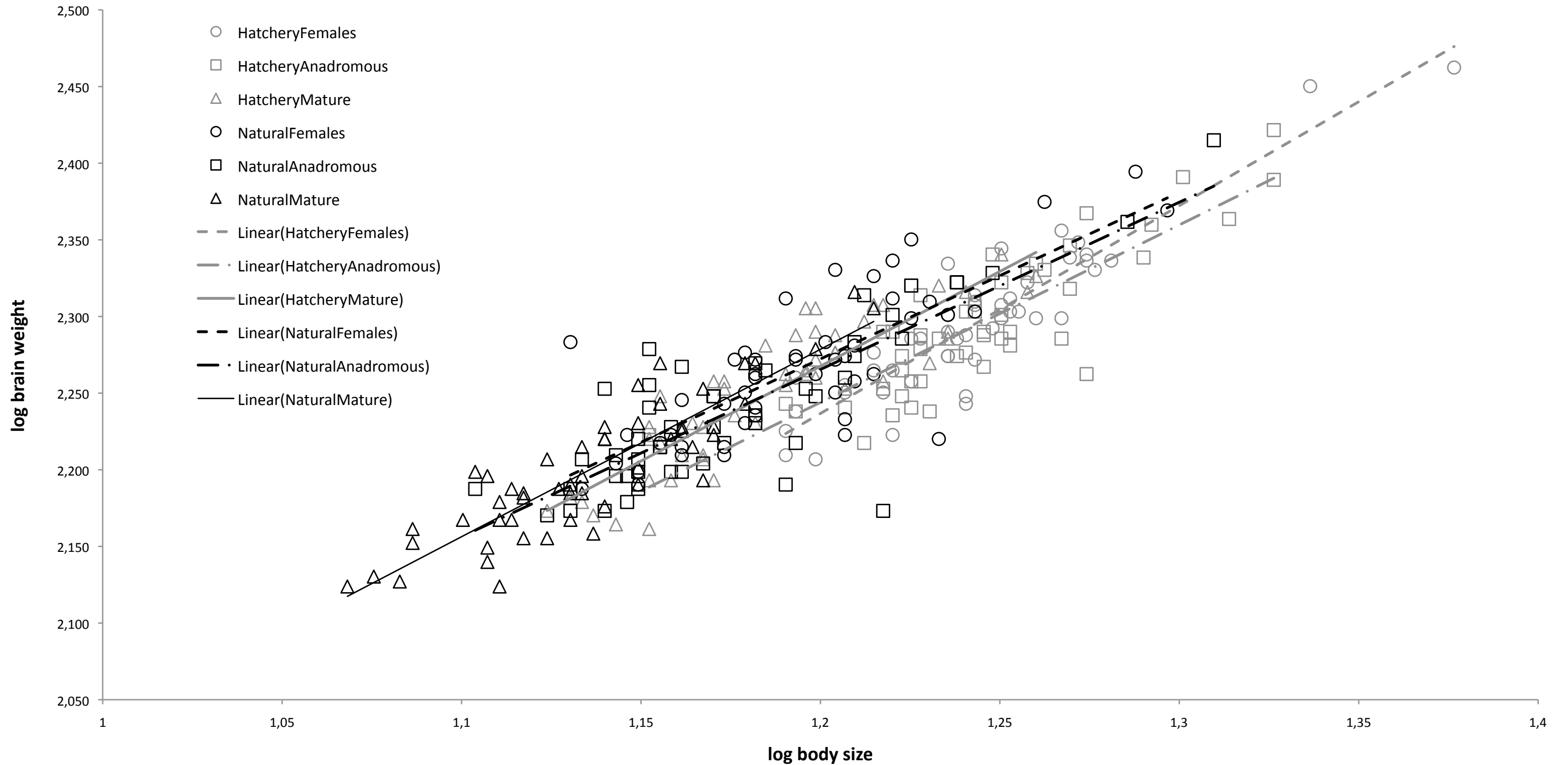

Supplement: Supplementary file 1 — Supplementary material 1 (PDF 42 kb) [file 10682_2014_9715_MOESM1_ESM.pdf]
